# Evidence for the homogeneous ferromagnetic phase in (Ga,Mn)(Bi,As) epitaxial layers from muon spin relaxation spectroscopy

**DOI:** 10.1038/s41598-019-40309-y

**Published:** 2019-03-04

**Authors:** K. Levchenko, T. Prokscha, J. Sadowski, I. Radelytskyi, R. Jakiela, M. Trzyna, T. Andrearczyk, T. Figielski, T. Wosinski

**Affiliations:** 1 Institute of Physics, Polish Academy of Sciences, Aleja Lotnikow 32/46, PL-02668 Warsaw, Poland; 2 Paul Scherrer Institute, Laboratory for Muon Spin Spectroscopy, CH-5232 Villigen, Switzerland; 3 MAX-IV Laboratory, Lund University, P.O. Box 118, SE-221 00 Lund, Sweden; 4 Department of Physics and Electrical Engineering, Linnaeus University, SE-391 82 Kalmar, Sweden; 5 Faculty of Mathematics and Natural Sciences, University of Rzeszów, PL-35310 Rzeszów, Poland

## Abstract

Ferromagnetic semiconductor thin layers of the quaternary (Ga,Mn)(Bi,As) and reference, ternary (Ga,Mn)As compounds, epitaxially grown under either compressive or tensile strain, have been characterized from a perspective of structural and magnetization homogeneity. The quality and composition of the layers have been confirmed by secondary-ion mass spectrometry (SIMS). A thorough evaluation of the magnetic properties as a function of temperature and applied magnetic field has been performed by means of SQUID magnetometry and low-energy muon spin relaxation (µSR) spectroscopy, which enables studying local (on the nanometer scale) magnetic properties of the layers. The results testify that the ferromagnetic order builds up almost homogeneously below the Curie temperature in the full volume fraction of both the (Ga,Mn)As and (Ga,Mn)(Bi,As) layers. Incorporation of a small amount of heavy Bi atoms into (Ga,Mn)As, which distinctly enhances the strength of spin-orbit coupling in the quaternary (Ga,Mn)(Bi,As) layers, does not deteriorate noticeably their magnetic properties.

## Introduction

The coexistence of semiconducting and ferromagnetic properties in dilute ferromagnetic semiconductors (DFS) moved from being a concept idea among many possible ones to basically one of the main progress vectors to succeed in spin electronics, usually referred to as spintronics^1^. Interaction between localized magnetic moments and charge carriers (holes in the case of (Ga,Mn)As) creates a wide variety of possible applications for this canonical DFS^2,3^. Several concepts of magneto-resistive memory devices, based on the interplay of intrinsic properties and extrinsic fabrication of (Ga,Mn)As thin layers have recently been proposed. One of them makes use of a large magnitude of the planar Hall effect (PHE) in (Ga,Mn)As^4–7^, which results from the combined effects of strong spin-orbit interaction in the valence band of the crystal with zinc blende structure and the large spin polarization of holes below the ferromagnetic phase transition Curie temperature, *T*_*C*_. Another one takes advantage of a competition between two types of magnetic anisotropies: the crystalline anisotropy of (Ga,Mn)As thin layer and the patterning-induced (shape) anisotropy in nanostructures of various geometries fabricated from the layer by means of electron-beam lithography and chemical etching^8–10^. Both concepts allow achieving new type of non-volatile memory elements, in which a bit of information can be written magnetically and read electrically. The output signal in such devices can be increased by addition of a small fraction of bismuth into (Ga,Mn)As, which enhances the magneto-transport effects in the quaternary (Ga,Mn)(Bi,As) compound, as demonstrated recently^11,12^. This is caused by a strong enhancement of the strength of spin-orbit coupling resulting from the replacement of As atoms by much heavier Bi atoms, leading to a large relativistic correction to the GaAs band structure^13,14^. First reports on magnetic properties of (Ga,Mn)(Bi,As) epitaxial layers displayed their similarity to those of (Ga,Mn)As ones with somewhat lower *T*_*C*_^12,15^.

However, inevitable practical limitations can appear stemming from the non- homogeneity of the material. The magnetic memory concept study and test measurements were done while keeping in mind a structural homogeneity of the DFS thin films and uniform magnetisation (one domain) in corresponding areas. In order to predict, control and reveal the full potential of the structure, a series of thorough measurements are required to prove the feasibility for practical applications.

In the present paper we report on magnetic characterization of the (Ga,Mn)(Bi,As) and reference (Ga,Mn)As epitaxial layers grown under either compressive or tensile strain. The magnetic properties of the DFS layers have been studied by magnetization measurements with the superconducting quantum interference device (SQUID) magnetometry and by using the low-energy muon spin relaxation (LE-µSR) spectroscopy to determine the magnetic volume fractions (i.e. spatial magnetic homogeneity), performed on the same set of samples. Composition of the layers has been examined with the use of secondary-ion mass spectrometry (SIMS). The high structural perfection of the layers and good quality of their interfaces were confirmed in our previous high-resolution X-Ray diffraction (HR-XRD) measurements^16^.

## Samples and Experiments

(Ga,Mn)(Bi,As) layers with 6% Mn and 1% Bi contents and 50 nm thickness were grown by the low-temperature molecular-beam epitaxy (LT-MBE) technique at a temperature of about 230 °C on either semi-insulating (001)-oriented GaAs substrate or the same substrate covered with a 0.63-μm thick, strain relaxed In_0.2_Ga_0.8_As buffer. Reference (Ga,Mn)As layers with 6% Mn content were grown under the same conditions. The growth conditions were optimized, as described in ref.^17^, to reduce the concentrations of arsenic antisite and interstitial Mn defects in the layers. As proved by the HR-XRD examination^16^, all the layers were pseudomorphically strained either to the GaAs substrate or to (In,Ga)As buffer under compressive or tensile strain, respectively. After the growth the layers were subjected to a low-temperature annealing treatment performed in air at the temperature of 180 °C during 50 h to improve their structural quality and magnetic properties^15,18^. Annealing thin (Ga,Mn)As layers at temperatures below the growth temperature results mainly in out-diffusion of Mn interstitials, causing reduction in the hole concentration and magnetic moment in the as-grown layers^19,20^.

Four wafers of the surface area of about 1 cm^2^, containing either the (Ga,Mn)(Bi,As) or (Ga,Mn)As layers grown under compressive and tensile misfit strain, have been subjected to the LE-µSR spectroscopy measurements. Next, small samples, of the area of 0.15 cm^2^ approximately, cleaved from the same wafers have been examined by means of the SQUID magnetometry and SIMS analysis.

## Experimental Results and Discussion

### SIMS analysis and SQUID magnetometry

SIMS analysis has been carried out to determine precisely the in-depth composition of the corresponding layers in the investigated samples. The detailed results of SIMS measurements, using a Cameca IMS 6 F micro-analyser with the cesium primary beam, are shown in Fig. S1 in Supplementary Material. The depth profiles confirm the uniform distribution of all the elements in the layers and 50-nm thickness of the Mn-containing top DFS layers. Moreover, they quantitatively confirm the Mn content in those layers (previously calibrated during the MBE growth) within the accuracy of the technique.

**Figure 1:**
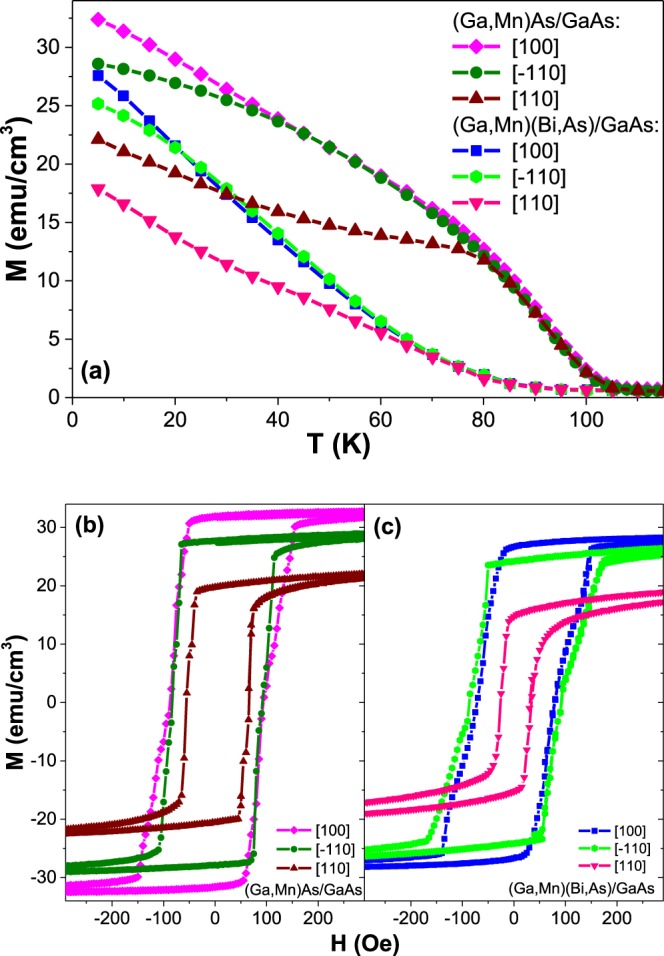
SQUID magnetometry results for the (Ga,Mn)As and (Ga,Mn)(Bi,As) layers grown under compressive strain on GaAs substrate measured under a magnetic field along the main in-plane crystallographic directions denoted in the figure: (**a**) magnetization vs. temperature under a magnetic field of 100 Oe, (**b**,**c**) magnetization hysteresis loops at a temperature of 5 K. Diamagnetic contributions from the substrate have been subtracted.

The temperature- and field-dependent SQUID magnetometry measurements, under a magnetic field applied along the main in-plane crystallographic directions and the direction perpendicular to the growth plane, have been performed for the same set of samples in order to characterize the macroscopic magnetization behaviour of the ternary and quaternary compounds and their magneto-crystalline anisotropy. The results obtained for the layers grown under compressive misfit strain on GaAs substrate measured under in-plane [100], $[\bar{1}10]$ and [110] crystallographic directions are presented in Fig. 1. The magnetization hysteresis loops measured at a temperature of 5 K, shown in Fig. 1b,c, clearly indicate that, for both the (Ga,Mn)As and (Ga,Mn)(Bi,As) layers, easy magnetization axes are oriented along the in-plane 〈100〉 cubic directions and hard axes along two magnetically non-equivalent in-plane 〈110〉 directions (so-called uniaxial anisotropy). Such behaviour, with the $[\bar{1}10]$ direction being magnetically easier than the perpendicular [110] one, is characteristic of compressively strained (Ga,Mn)As layers with high concentration of valence-band holes^9,21^. The origin of the uniaxial in-plane anisotropy is still debated^22–24^ and most probably results from the preferred formation of Mn dimmers along the $[\bar{1}10]$ crystallographic direction at the (001) surface during the epitaxial growth of (Ga,Mn)As layers, as concluded from the recent *ab initio* calculations^25^. The (Ga,Mn)As and (Ga,Mn)(Bi,As) layers are characterized by the *T*_*C*_ values of about 105 K and 90 K, respectively, as evaluated from the temperature dependences of SQUID magnetization shown in Fig. 1a.

**Figure 2:**
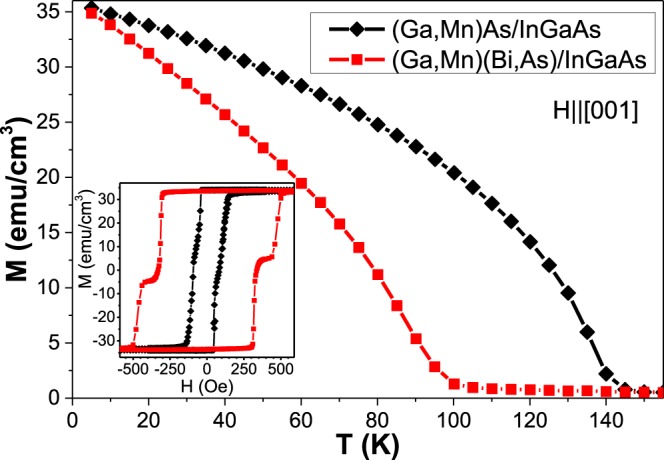
SQUID magnetization vs. temperature for the (Ga,Mn)As and (Ga,Mn)(Bi,As) layers grown under tensile strain on (In,Ga)As buffer measured under a magnetic field of 100 Oe along the out-of-plane [001] crystallographic direction. Inset: magnetization hysteresis loops at a temperature of 5 K. Diamagnetic contributions from the substrate and buffer have been subtracted.

The (Ga,Mn)As and (Ga,Mn)(Bi,As) layers, grown under tensile misfit strain on the relaxed (In,Ga)As buffer layer with larger lattice parameter, have the easy magnetization axis oriented along the [001] growth direction, typical of tensile-strained (Ga,Mn)As layers^26,27^. The SQUID magnetometry results obtained for those layers under a magnetic field applied along the growth direction, shown in Fig. 2, reveal the *T*_*C*_ values of about 145 K and 100 K, respectively. The magnetization hysteresis loops for those layers, measured at a temperature of 5 K, are shown in the inset in Fig. 2. Intriguingly, the tensile-strained (Ga,Mn)(Bi,As) layer exhibits much broader hysteresis loop with respect to that of the compressively strained one and those of the (Ga,Mn)As layers. Similar results, indicating distinctly larger coercive fields for the tensile-strained (Ga,Mn)(Bi,As) layers, were obtained by means of magneto-optical Kerr effect magnetometry applied to thinner, 15-nm thick, layers^16^. Moreover, the step-like hysteresis loop shown in the inset in Fig. 2 for the (Ga,Mn)(Bi,As) layer may suggest the presence of ferromagnetic domains with different coercive fields and a multidomain magnetization reversal in the layer. Such multidomain magnetization reversal was previously observed with planar Hall effect measurements for (Ga,Mn)As layers^6^ and (Ga,Mn)As/GaAs superlattices^7^.

### Muon spin relaxation spectroscopy results and analysis

The SQUID magnetometry is a common technique used for investigation of the macroscopic magnetisation of magnetic materials. On the other hand, the µSR spectroscopy gives information about local magnetic field, thus is a perfect tool for probing the homogeneity of magnetically ordered volumes. We investigated our DFS layers by µSR spectroscopy using the low-energy muon beam facility at the Paul Scherrer Institute, Switzerland (LE-µSR setup LEM at the µE4 surface muon beam line^28–30^). At LEM nearly 100% polarized positive muons, µ^+^, are available with tuneable energies between 1 and 30 keV, which correspond to the mean implantation depths of a few nm up to 200 nm in GaAs. After implantation, the muons stop at interstitial lattice sites in the sample and decay with a lifetime of 2.2 µs, emitting decay positrons preferentially in the direction of muon’s spin. The probability of positron emission, *W*, as a function of the angle between the positron trajectory and the muon spin, *θ*, is given by the dependence: $W(\theta )d\theta \propto (1+A\,\cos \,\theta )d\theta$, where *A* is the asymmetry parameter averaged over all positron energies. In the presence of a magnetic field at the muon stopping site the muon spin precesses around the field. By detecting the emitted decay positrons with several detectors surrounding the sample the time evolution of the muon spin polarization can be determined. A typical µSR asymmetry spectrum registered with a pair of opposite positron detectors together with the Gaussian depolarization fitting curve is presented in Fig. S2 in Supplementary Material.

By applying a weak magnetic field of several tens Oe, transverse to the initial muon spin direction, the muon spin will precess at its Larmor frequency in a non-magnetic sample. This precession manifests itself as oscillations on the muon’s decay curve whose amplitude is given by the decay asymmetry in positron emission. If muons are implanted into a ferromagnetic sample, the internal large magnetic field – compared to the externally applied weak magnetic field – causes a very quick loss of muon spin polarization and vanishing muon decay asymmetry. This enables recognizing the transition from the paramagnetic to the ferromagnetic state and determining the fraction of sample volume turning into the ferromagnetic state. In case of a homogenous magnetization (i.e. 100% magnetic volume fraction) the observable decay asymmetry of the transverse field precession will drop to zero. Since the precession amplitude, stemming from asymmetric emission of positrons, is the principal quantity carrying interesting information about material, a proper care should be taken to correctly determine this quantity. For the analysis of the data we have used the program musrfit^31^.

Two previous studies of ferromagnetic (Ga,Mn)As layers performed by other research groups with the µSR spectroscopy at LEM facility showed discrepant results. The layers displayed either a spatial magnetic inhomogeneity, with ferromagnetic and paramagnetic regions^32^, or a homogeneous ferromagnetic order with a 100% magnetic volume fraction and a rather sharp onset of the ordering at *T*_*C*_^33^, depending on the layer preparation and annealing procedures. Later on, the µSR spectroscopy has also been successfully applied to examine the ferromagnetic volume fraction in other dilute magnetic semiconductors, such as Li(Zn,Mn)As^34^ and (La,Ba)(Zn,Mn)AsO^35^ bulk crystals and Co-doped TiO_2_^36^ thin films.

In the present experiments, the investigated samples, containing (Ga,Mn)As and (Ga,Mn)(Bi,As) layers, were glued with a silver paste onto a Ni coated sample plate on a cold finger cryostat. About 50% of the muons stopped in the samples (muon beamspot has a full-width-at-half-maximum of about 12 mm). Muons missing the sample and stopping in the Ni backing are quickly depolarized due to the presence of large internal magnetic fields inside the ferromagnetic Ni layer and do not contribute to the precession. Moreover, at low energies of implanted muons, of below 5 keV, a fraction of muons is being reflected in the decelerating electric field in front of the sample. Some of the reflected muons stop in the radiation shield of the sample cryostat, where they experience about the same magnetic field as the muons stopping in the sample. These muons create an additional false signal not originating from the sample. This false signal was measured, in dependence of the muon energy, using a Ni coated sample plate. Obtained results have been confirmed by using the program musrSim, a Geant4 based beam transport and muon spectrometer simulation^37,38^. This false signal has been subtracted from the measured one to obtain the decay asymmetry originating from muons stopping in the sample, further labelled “corrected asymmetry”, *A*_corr_.

**Figure 3:**
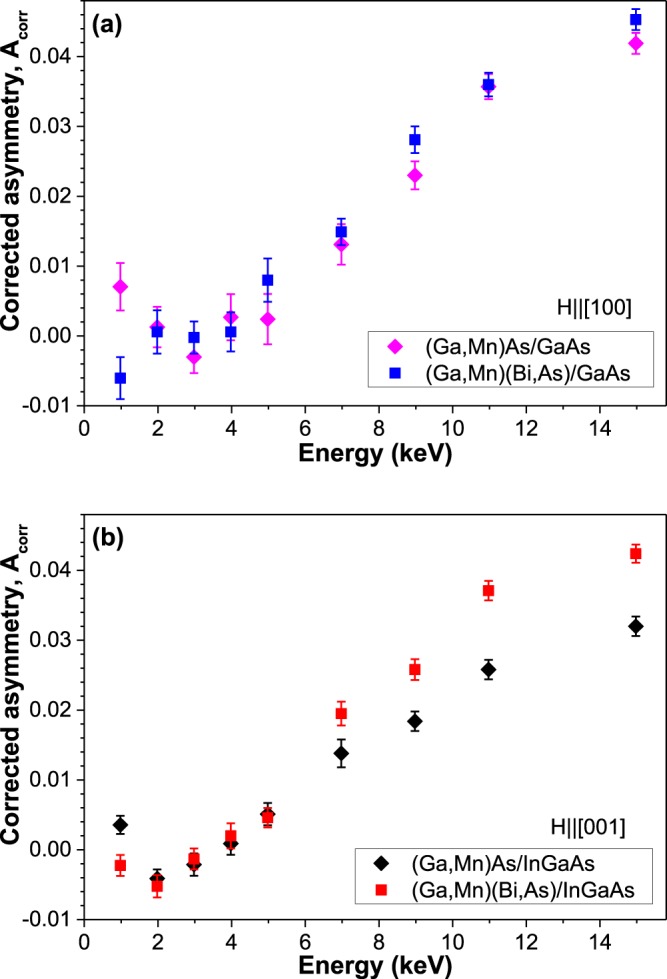
Comparison of the corrected µSR asymmetry (in arbitrary units) as a function of muon implantation energy obtained at a temperature of 5 K, under a weak magnetic field of 75 Oe, applied prior to cooling down, along the DMS easy magnetization axis; for the (Ga,Mn)As and (Ga,Mn)(Bi,As) layers grown on GaAs substrate, field applied along the in-plane [100] direction, (**a**) and for the layers grown on (In,Ga)As buffer, field applied along the out-of-plane [001] direction, (**b**).

**Figure 4:**
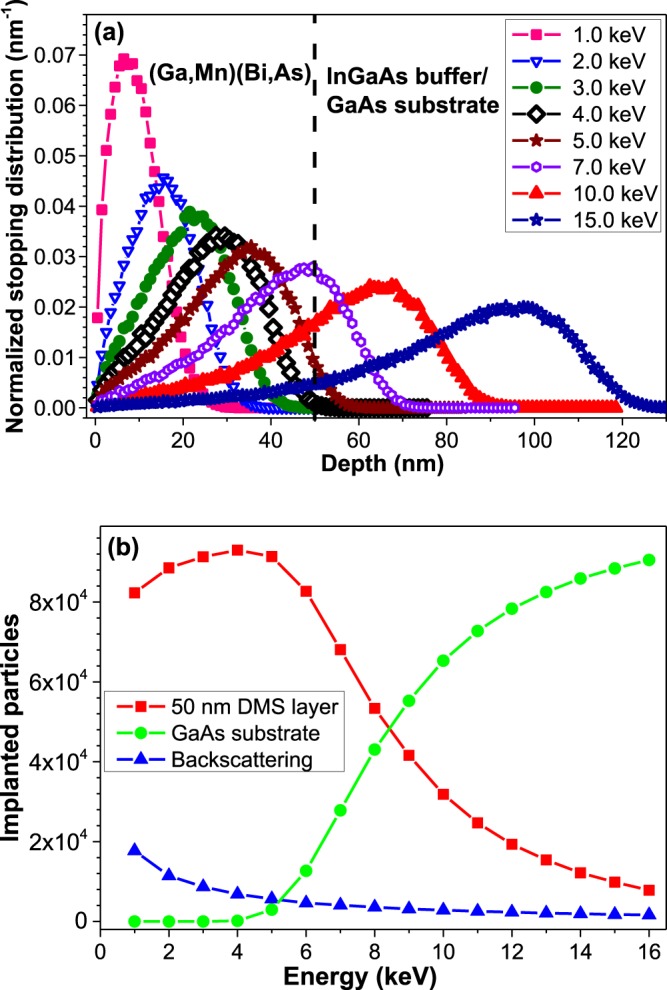
Calculated muon stopping profiles for various implantation energies denoted in the figure (**a**) and number of muons stopped in the top 50-nm-thick DMS layer, in the GaAs substrate layer, and backscattered muons (**b**). Stopping profiles have been calculated with the program TrimSP^39,40^.

Energy dependences of the corrected asymmetry, *A*_*corr*_, measured on our samples at a temperature of 5 K under a weak magnetic field of 75 Oe applied transverse to the initial muon spin direction, are shown in Fig. 3. Two different magnets on the LEM spectrometer were used in order to apply a magnetic field along one of the easy magnetization axes of investigated DFS layers (i.e., the in-plane [100] crystallographic direction for the compressively strained layers grown on GaAs substrate, shown in Fig. 3a, and the out-of-plane [001] direction for the tensile-strained ones grown on InGaAs buffer, shown in Fig. 3b). For comparison, the muon stopping profiles and stopped fractions as a function of the muon implantation energy calculated with the program TrimSP^39,40^ are shown in Fig. 4.

As can be seen in Fig. 3, for all the samples we obtained *A*_*corr*_ ~ 0 up to the muon implantation energies of 4 keV, i.e. where all of the non-reflected muons stopped in the DFS layer. This indicates a homogeneous ferromagnetic phase with a nearly 100% volume fraction. At the energy of 5 keV muons start to penetrate into the non-magnetic substrate, which leads to the observed increase in *A*_*corr*_, in accordance with the calculated results shown in Fig. 4, thus confirming the thickness of 50 nm of the DFS top-layers. Additionally, in Fig. S3 in Supplementary Material we present the Gaussian depolarization rate as a function of muon implantation energy, obtained for the investigated samples for the energies ≥7 keV where contributions from reflected muons stopping in the radiation shield can be neglected. In the bulk of GaAs the depolarization rate of μ^+^ in a weak transverse field is typically ~0.18 µs^−1^ due to the nuclear dipolar fields of the Ga and As nuclei. On approaching the interface with the DFS layer, the depolarization rate increases by a factor of 3 to 5 due to the magnetic stray fields from the ferromagnetic layer. This rather weak stray field contribution further supports the picture of a single domain, homogenous ferromagnetic phase and it indicates a smooth interface with very low roughness of the order of 1 nm^41,42^.

**Figure 5:**
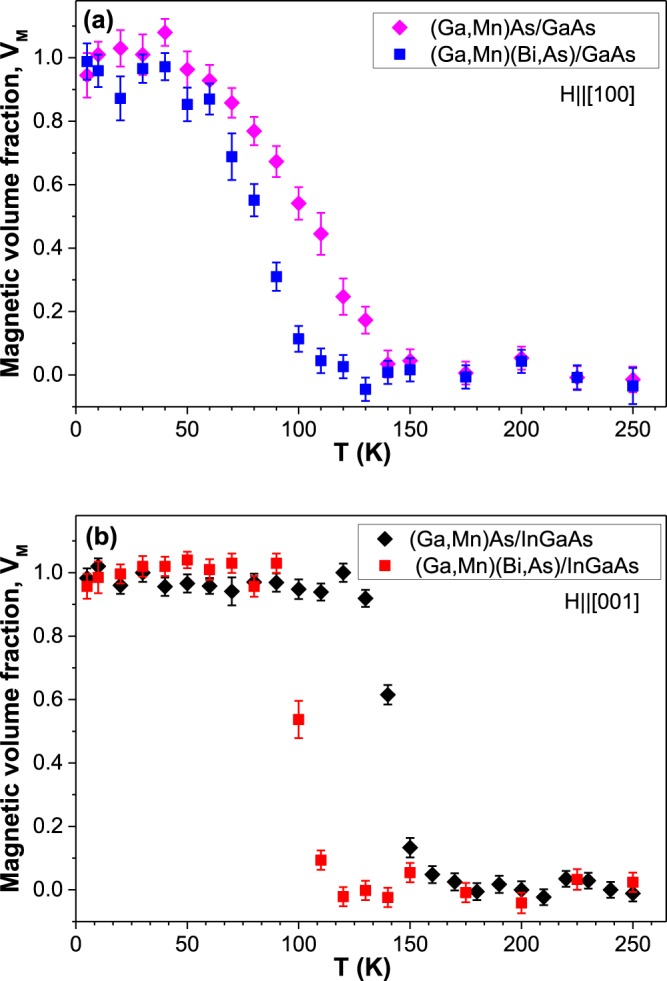
Magnetic volume fraction *V*_*M*_, defined with Eq. (1), as a function of temperature, measured under the same magnetic fields as in Fig. 3, for the (Ga,Mn)As and (Ga,Mn)(Bi,As) layers grown on GaAs substrate (**a**) and for the layers grown on (In,Ga)As buffer (**b**).

For measurements of the magnetic volume fraction, *V*_*M*_, as a function of temperature we have chosen the muon implantation energy of 4 keV, at which most of the muons stop in the centre of the top 50-nm-thick DFS layer. The results for all the four samples are shown in Fig. 5, where *V*_*M*_(*T*) was estimated as:

1 $${V}_{M}(T)=1-\frac{{A}_{corr}(T)}{{A}_{corr}(T\ge {T}_{C})}$$

where *T* is temperature, *A*_*corr*_(*T*) is the temperature dependent corrected asymmetry parameter and *A*_*corr*_(*T* ≥ *T*_*C*_) is the corrected asymmetry in the paramagnetic phase above *T*_*C*_.

From Fig. 5b we see that the transitions to ferromagnetic state with decreasing temperature in the tensile-strained DFS layers grown on the InGaAs buffer is rather sharp, and *V*_*M*_ reaches about unity within 10 K. On the other hand, in the compressively strained layers grown on the GaAs substrate the onset of the transitions is much broader, and *V*_*M*_ reaches about unity within 60–80 K (Fig. 5a). The latter more gradual increase in *V*_*M*_ on lowering temperature may result from the magnetic easy axis reorientations as a function of temperature in the compressively strained layers, which have been experimentally observed and theoretically justified for (Ga,Mn)As layers^21,43,44^. Importantly, in all the investigated DFS layers a homogeneous ferromagnetic order with about 100% magnetic volume fraction is observed at low temperature in contradiction to the previous results by Storchak *et al*.^32^ obtained for their (Ga,Mn)As epitaxial layers.

In the µSR measurements the Curie temperature corresponds to the temperature, at which *V*_*M*_ = 0.5^45^. Thus, the *T*_*C*_ values obtained from the results shown in Fig. 5 are about 100 K and 85 K for the compressively strained (Ga,Mn)As and (Ga,Mn)(Bi,As) layers, respectively, and 140 K and 100 K for the tensile-strained (Ga,Mn)As and (Ga,Mn)(Bi,As) ones, respectively. These *T*_*C*_ values agree fairly well with those of the onset of magnetization obtained from the SQUID magnetometry measurements.

## Conclusions

High-quality layers of the quaternary dilute magnetic semiconductor (Ga,Mn)(Bi,As), together with the reference layers of canonical (Ga,Mn)As DFS, have been prepared by means of the LT-MBE growth and post-growth annealing. Thorough SQUID magnetometry examination of the layers, grown both under compressive and tensile misfit strain, revealed similar magnetic properties of the two compounds with rather complicated magneto-crystalline anisotropy induced by the lattice strain present in the layers, and the Curie temperature of the quaternary compound being consistently lower than that of the ternary one.

Spatially resolved low-energy muon spin relaxation spectroscopy investigations, performed as a function of both the muon implantation energy and temperature, demonstrate that a homogeneous long-range ferromagnetic spin ordering develops below the Curie temperature in almost full volume fraction in all the investigated DFS layers. The obtained results also suggest the appearance of a single ferromagnetic domain in the layers under a weak magnetic field of 75 Oe and very smooth interfaces between the layers and the buffer or substrate. The (Ga,Mn)(Bi,As) layers, where incorporation of a small amount of heavy Bi atoms into (Ga,Mn)As DFS layers results in a strong increase in the strength of spin-orbit coupling and, consequently, enhancement of the magnitude of magneto-electric phenomena, may be especially favourable for specific spintronic functionalities utilizing electrically controlled spin polarization of charge carriers.

## Supplementary information

Supplementary Material

## Data Availability

All data generated or analysed during this study are included in this published article and its Supplementary Material files.
