# Supplementary material for: Evidence for the homogeneous ferromagnetic phase in (Ga,Mn)(Bi,As) epitaxial layers from muon spin relaxation spectroscopy
Source: Sci Rep. 2019 Mar 4;9:3394. doi: 10.1038/s41598-019-40309-y (PMC6399229; doi:10.1038/s41598-019-40309-y)
Supplement: Supplementary file 1 — Supplementary Material [file 41598_2019_40309_MOESM1_ESM.doc]

**Supplementary Material for:**

**Evidence for the homogeneous ferromagnetic phase in (Ga,Mn)(Bi,As) epitaxial layers from muon spin relaxation spectroscopy**

**K. Levchenko^1^*****, T. Prokscha^2^, J. Sadowski^1,3,4^, I. Radelytskyi^1^, R. Jakieła^1^, M. Trzyna^1,5^, T. Andrearczyk^1^, T. Figielski^1^ and T.Wosinski^1^**

^1^ *Institute of Physics, Polish Academy of Sciences, Aleja Lotnikow 32/46, PL-02668 Warsaw, Poland*

^2^ *Paul Scherrer Institute, Laboratory for Muon Spin Spectroscopy, CH-5232 Villigen, Switzerland*

^3^ *MAX-IV Laboratory, Lund University, P.O. Box 118, SE-221 00 Lund, Sweden*

^4^ *Department of Physics and Electrical Engineering, Linnaeus University, SE-391 82 Kalmar, Sweden*

^5^ *Faculty of Mathematics and Natural Sciences, University of Rzeszów, PL-35310 Rzeszów, Poland*

*E-mail: levchenko@ifpan.edu.pl

Secondary-ion mass spectrometry (SIMS) results for the 50-nm thick (Ga,Mn)As and (Ga,Mn)(Bi,As) epitaxial layers grown either on GaAs substrate or on 0.63-μm thick In_0.2_Ga_0.8_As buffer are presented in Fig. S1.

**(a)**

**(b)**

**(c)**

**(d)**

Fig. S1. Depth profiles of Mn, Bi, In, Ga and As determined with SIMS for the investigated samples: (a) (Ga,Mn)/GaAs, (b) (Ga,Mn)(Bi,As)/GaAs, (c) (Ga,Mn)As/(In,Ga)As, and (d) (Ga,Mn)(Bi,As)/(In,Ga)As. The Mn concentration in atoms per volume unit is shown on the left vertical axis and the SIMS signals in counts per second for all the other elements are shown on the right vertical axis in all the panels.

Fig. S2 displays a typical muon spin relaxation (µSR) time spectrum measured with two positron detectors placed opposite to each other for the (Ga,Mn)(Bi,As) layer grown on (In,Ga)As. The asymmetry spectrum was obtained under external weak magnetic field of 75 Oe applied perpendicular to the initial muon spin polarization direction, at a temperature of 150 K (above the ferromagnetic transition temperature) and at a muon implantation energy of 4 keV (where the muons stopping distribution is centered in the middle of the DFS layer). The asymmetry spectrum demonstrates the time evolution of the polarization of muon spins precessing at the Larmor frequency of about 1 MHz. The asymmetry spectrum has been fitted with a single component cosine function, where the depolarization is described with the Gaussian function:

$A(t)=A_{0}\exp(-\frac{(\sigma t)^{2}}{2})\cos(\omega t+\phi)$,

where *σ* is the Gaussian depolarization rate, *t* is time, *ω* is frequency (depending on the local magnetic field *B_loc_*: $\omega=\gamma_{\mu}B_{loc}$, where $\gamma_{\mu}=2\pi\cdot135.5$ MHz/T is the muon gyromagnetic ratio) and *ϕ* is a phase correction depending on the geometry of positron detectors.

Fig. S2. Asymmetry spectrum of two positron detectors placed opposite to each other for the (Ga,Mn)(Bi,As) layer grown on (In,Ga)As, measured under external weak magnetic field of 75 Oe, at a temperature of 150 K and at a muon implantation energy of 4 keV. The fit is a single component cosine function with Gaussian depolarization, as described above.

Fig. S3 displays the Gaussian depolarization rate at the muon implantation energies ≥ 7 keV, where contributions from reflected muons stopping in the radiation shield can be neglected. In the bulk of GaAs the depolarization rate of μ^+^ in a weak transverse field is typically ~0.18 µs^-1^ due to the nuclear dipolar fields of the Ga and As nuclei. This value is approximated at the implantation energies ≥ 15 keV, where the mean distance of the stopped muons to the interface of the ferromagnetic layer is ≥ 40 nm (cf. Fig. 4 in the main text). On lowering the implantation energy, i.e. stopping the muons closer to the interface, the depolarization rate increases by a factor of 3-5 close to the interface. This increase can be attributed to magnetic stray fields from the ferromagnetic layer. The contribution of the stray fields to the depolarization is rather weak: the nuclear depolarization rate of 0.18 μs^-1^ deep in the GaAs substrate corresponds to a Gaussian width of about 2 Oe, which increases to 6-10 Oe close to the interface. This weak stray field effect and its disappearance within ~50 nm from the interface supports the picture of a single domain, homogeneous ferromagnetic phase, and it indicates a smooth interface with very low roughness of the order of 1 nm [1,2]. Note, that although the stray field distribution close to the interface is only approximated by the Gaussian depolarization used in our analysis, the fits as a function of energy with a Gaussian depolarization function describe well the data with reduced χ^2^ values very close to 1.

Fig. S3. Gaussian depolarization rate σ under a weak magnetic field of 75 Oe as a function of muon implantation energy at 5 K for the (Ga,Mn)As and (Ga,Mn)(Bi,As) layers grown under tensile strain on (In,Ga)As. For the layers grown on GaAs the depolarization rates agree within the experimental errors.

[1] Drew A.J., Hoppler J., Schulz L., Pratt F.L., Desai P., Shakya P., Kreouzis T., Gillin W.P., Suter A., Morley N.A., Malik V.K., Dubroka A., Kim K.W., Bouyanfif H., Bourqui F., Bernhard C., Scheuermann R., Nieuwenhuys G.J., Prokscha T., and Morenzoni E., *Nat. Mater.* **8**, 109 (2009).

[2] Tsymbal E., *J. Magn. Magn. Mater*. **130**, L6 (1994).
